# The Philosophical Treatment of Diphtheria

**Published:** 1879-11

**Authors:** Styles Kennedy


### THE PHILOSOPHICAL TREATMENT OF DIPHTHERIA.

By STYLES KENNEDY, M.D.

So much has been written on the treatment of diphtheria, that it almost seems necessary to give some reason for asking further attention to the subject just now, especially so as the writer has nothing original to offer. Although Michigan is fearfully ravaged by diphtheria, I have failed to find anywhere a philosophical treatment, a treatment based on the etiology and pathology of the disease, as exemplified by the Germans, Eberth, Nassiloff, Tomassi, Oertel and others. I shall not constantly pause to give authority and citations, but those who feel an interest in getting out of ceaseless and useless empiricism are referred to the splendid article of one hundred and twenty-five pages, on diphtheria by Dr. Oertel, in Ziemssen's recent work.

As is well known, diphtheria presents itself as a *local* and as a *general disease.* What explanation can be given of the diseased process itself? What are the relations of the local and general disease? Which causes the other? The view here maintained is that diphtheria is primarily a local disease. For obvious reasons this point must be settled by pathological experimentation. If diphtheritic poison be placed beneath the skin or mucous membrane, the first pathological changes occur at the *point of inoculation* as an inflammatory action, and leads to the formation of a grayish-white false membranous deposit. If diphtheria is a general infectious disease with secondary localizations, it would follow the law of that class of disease, and have a fixed point of localization, and always localize itself on the mucous membranes of the throat and air passages; just as the inoculation of horses with the poison of glanders always produces its effects locally upon the nasal mucous membrane, notwithstanding the poison is injected beneath the skin.

The microscope has revealed certain vegetable organisms in connection with diphtheria, and the important question is to determine the relations that exist between the organisms and the disease. This would seem to involve a discussion of the nature and character of the contagion. The vegetations are of exceeding minuteness, and consist principally of spherical bacteria (micrococcus,) accompanied by the bacteria termo. These occur constantly, and infest the false membranes and tissues underlying them. With increase of disease we find increase of these organisms in the infected parts. Just before a diphtheritic layer is formed, a great and rapid increase of micrococcus and bacterium termo takes place. Other forms of fungus, cryptococcus, leptotrix buccalis, etc., may be present on the advent of the forms here mentioned, but they are speedily destroyed. Frequently it is noticed, during the process of recovery, that the micrococcus and bacteria termo vanish, and the other vegetable forms predominate. In the first hours of the disease, before there is any distinction of tissue, the delicate, ring-shaped, greyish-white spots, scarcely rising above the level of the mucous membrane, consist solely of micrococcus growths pushing out the cells of different epithelial layers. If various bacteria besides the micrococcus be present in the inoculated matter, it is noticed that only the micrococci and bacterium termo have prolific growth, while the other forms disappear entirely. It is interesting in this connection to know that if the inoculating material be filtered or agitated so as to separate the micrococci and bacterium termo from the fluid placed under the skin, no inoculation takes place. There is no doubt about these organisms being inseparable from the diphtheritic process. *Without micrococci there can be no diphtheria*, and the intensity of the toxic infection increases with their increase.

In the lighter forms of diphtheria the anatomico-pathological changes that take place in the cavity of the mouth and pharynx during the first twelve or eighteen hours are those of simple catarrhal inflammation. Very soon appears the witish-gray, hoar-frost-like coating, gradually developing into the more yellow or dirty-green layers. The agent, in altering the color and size of the parts, is the micrococcus accumulating in the epithelia layers. Slowly the micrococcus forces its way down into deeper layers, forcing superficial layers and deposits above the general surface. A few hours later pus-corpuscles appear in the deeper layers; they gradually infiltrate the sub-epithelial and circumscribe the fungus growth. With the pus corpuscles come the large young cells with large nuclei and active proliferation. Then streams and masses of pus corpuscles, as also the cells, form broad lines of demarkation against the growth of the fungus. Finally, the widening masses of pus and young epithelial cells force the more or less extensive witish-gray patch from its attachments, leaving a red and injected place, to be rapidly covered by renewed epithelial layers, and the disease ends in a few days.

In the croupous form of diphtheria, the characteristic sign is exudation of fibrine, and its peculiar network is seen in the patches. Fibrine is poured out into the epithelium, and into the interstices between it and the sub-epithelial tissue. In a few hours the surface of the false membrane is seen to be in process of necrosis and fibrinous degeneration. The micrococcus penetrates deeper and deeper, new deposits of fibrine continue to be poured out, forming in the superficial layers, broad, distinct, interlacing bands, while a fine network is found in the sub-epithelial tissue. The false membranes increase in all of their dimensions until death occurs, or, in favorable cases, the fine network is no further strengthened by fibrinous exudations, and the diseased mass is forced off by a new formation of pus and cells.

Or again, the pus-corpuscles and young cells make their way to the surface through the meshes and framework of the false membrane which has hitherto been free from them, causing the slow wearing away of the membrane by ulceration.

The anatomico-pathological changes in the septic form of diphtheria are not especially different from those described. Extensive decomposition and putrefaction, with membranes of dirty-gray, dark-brown color, infiltrated by capillary hemorrhage. The local infection having poisoned the system, the system reacts upon the local disorder, intensifying it in all directions. Occasionally, septicæmia does not produce death, till the intensity of the local infection by its mere power of destruction, or by mechanical pressure and arrest of nutrition, produce gangrene of the tissue. Now false membrane, mucosa, sub-mucosa, form a vast slough of a dark, worm-wood-like pulp, from which the intense, peculiar odor of gangrene is spread. The septic form most frequently follows diphtheria of the nose. In diphtheria of the larynx and trachea in children the free exudation of fibrine causes death by suffocation before septicæmia can take place.

We are now brought to face this fact: We have to treat a specific exudative inflammation of the mucous membrane and a general poisoning of the system. When left to the unaided efforts of nature, the false membranes become detached, and the urgent symptoms are abated through suppuration. The entrance of micrococci and the absorption of putrefying substances is prevented by a thick, impermeable layer of pus corpuscles. The process of healing in the case of wounds which are the seat of the diptheretic infection, is accomplished in the same way.

Prominent among antiphlogistic remedies is the abstraction of blood, but diphtheria of the mouth and throat is not in the least altered by the withdrawal of blood locally, while failure of strength may be hastened, and diptheretic ulcers may be created. Of course general blood-letting is out of the question.

Some years since I recommended to the profession the local application of ice to the sub-maxillary region in diphtheritic inflammation of the mucous membrane of the mouth and throat. I had used ice in this way in the inflammation of the throat in scarlet fever with the best of results, and reasoning by analogy, as I supposed, I thought it would do as well in diphtheria, and in actual practice I found the subjective symptoms of the inflammation were ameliorated. So distinguished a practitioner as Dr. Hiram Carson, of Pennsylvania, advocated the same practice. At that time, however, the micrococcus and bacteria termo had not been discovered as the specific influences of diphtheritic inflammation. Now we know that cold has no distinctive effect upon these vegetations, for after being kept at a temperature below zero for twenty-four hours, they again show themselves capable of motion and propagation. Ice will not check the spread, nor detach the false membrane, nor alter its histological composition On the other hand, under the employment of ice, fibrinous secretion keeps going on, while purulent infection cannot occur, and no purulent layer of demarkation and detachment takes place, while the pain relieved by the ice stands in no sort of relation to the severity of the disease.

One of the most popular methods of treatment has been, and still is to a large extent, by means of cauterization. Cauterization will not completely destroy the disease, because it is impossible, even with the greatest care and repeated operations, to bring the remedy in contact with the whole of the contagious material. The micrococcus is not only in the false membrane or grayish-white spots, but in the subjacent tissues and in the mucus and saliva of the mouth. No amount of care in the act of cauterization will prevent a certain degree of mechanical violence to the inflamed mucous membrane, and sub-epithelial membrane. These minute lacerations, scarcely if at all visible to the naked eye, are just so many new homes for the micrococcus. It is equally futile to try and convert the specific inflammation into a simple one by this means. There is no longer a single decent hypothesis upon which to base this miserable treatment. For these reasons, in part, the mechanical detachment of the false membranes, as being sources of inflammation, is no longer to be thought of. This is rather a summary way of disposing of a favorite method, but it must go. It may be occasionally necessary to interfere mechanically and remove false membrane to save the patient from suffocation, and only then. The result is not only the rapid reproduction of more false membrane, an increase of fibrinous exudation, but the spread of the disease, and, in the vast majority of cases, death from general infection. Death from suffocation is scarcely to be feared when the throat alone is affected, but only when the larynx and trachea are involved at the same time.

It is much better to attempt to relieve encroachment upon the air passages by dissolving the false membrane chemically. Here all the virtues of this means of treatment cease, for the chemical solution of the false membrance will have not the slightest influence on the affection of the mucous membrane. A new false membrane will form again and again. The danger of general infection is diminished.

On a false theory, it has for a long while been thought possible to antagonize the diseased processes by the local application of astringents. No diminution of the exudation can be obtained by this means, and if this was possible, nothing would be gained towards the cure of the inflammation and decomposition, nor towards the detachment of the false membrane, nor the destruction of the micrococci.

The method of treatment now to be described is simply an imitation of nature, or an assistant to nature in the effort to produce a sufficient supply of pus to form a barrier between the healthy and diseased tissues. This is attained by the employment of moist warmth, in the form of hot vapor, maintaining at short intervals a temperature of 118° to 122° F. in the mouth.

"The first appearances which are observed as a result of the operation of hot vapor are always constant and distinctly noticeable as early as at the end of from twelve to eighteen hours, during which the inhalation has been practiced hourly or half hourly for a quarter of an hour at a time; but these effects will be developed more slowly if a considerable fibrinous exudation, with partial decomposition of the pseudo-membranes, has already taken place, and the capacity for reaction of the tissues is almost extinguished; or they will not be produced at all when the disease has already run into septicæmia. The margins of the diphtheritic deposits, which generally pass imperceptibly into the surrounding tissues, become more sharply defined and contrast strikingly with the intensely reddened mucous membrane. The membranes, therefore, at the first glance seem enlarged. In some places, too, it will appear as if new membrane had formed where before there had been none; this is due to the fact that they had previously escaped notice from their small size and the presence of mucus which concealed their outlines. Thus it will appear as if the disease had increased in intensity. The operation of the hot vapor, however, has been to induce a considerable excretion of pus corpuscles, and these have infiltrated the epithelium, or its delicate net-work, which was already infected and grown full of micrococci. Under longer continued operation of the hot steam, soon no further enlargement of the patches will be noticed. The pseudo-membranes become gradually thicker, are raised up from the mucous membrane; their whitish-gray color becomes more yellowish or of a dirty gray, and their surfaces wrinkled and uneven, while the redness of the adjacent mucous membrane also fades and the swelling disappears. After some days we obtain, with the necessary amount of suppuration, a complete detachment of the pseudo-membranes, and they are expectorated by the patient, either whole or in single, scarcely noticeable fragments, or are, possibly, in part swallowed."

The successful use of hot vapor requires no little patience and some manipulative skill on the part of the physician and attendant; a want of these qualifications too often causes an abandonment of this plan of treatment before it has been half tried. I use a tin vessel from eight to twelve inches in diameter, with strong handle, the vessel being three to five inches high, with a well fitting cover. The top of the cover has a hole in it nearly two inches in diameter, around which is soldered a short tin collar, say two inches high. This vessel, properly filled with hot water, is set over an alcohol lamp, and the steam is conducted to the patient's face by means of two inch tin piping, such as is used for "conductors" on small houses. Two or three "elbows" of same size, and a few pieces of pipe, will be all that is necessary in this line. The pieces of pipe are easily fitted to each other, or the elbows. In cold weather the heating stove and tea kettle may be used with the tin pipe. Arrange the apparatus so that the end of the tube near the face will be a little lower than the face, as the steam will rise, and the patient not be on a strain to reach it. Sometimes a large cone made with a newspaper, the apex over the tube and the base covering the face, will facilitate matters. There is no necessity of scalding the patient's face, and only carelessness will let the patient use vapor below 110° Fah. The patient or attendant should use a thermometer for a while until they get the run of the business before them.

"With any suitable apparatus we can at the same time accomplish a thorough cleansing of the mouth and throat from mucus and the fluid which dissolves the mucus, but which, at the same time, acts indifferently on the organism. Such a fluid, steadily flowing over the mucous membrane, washes away masses of mucus, remnants of food and other products of decomposition. We can also use solution of chloride of sodium, or chlorate of potash, or other alkalies, only we must avoid strong disinfecting substances, such as carbolic acid or permanganate of potash, because, after long continued inhalation, more or less of these substances is always carried into the bronchi and may produce symptoms of irritation. Solution of common salt, or chlorate of potash, if this latter be preferred, of the strength of from ten to fifteen grains to the ounce, produce no injurious effect upon the organism, that is, they act perfectly indifferently when so used. How long these operations should last and how often they should be repeated must be determined by the degree of the affection, and it should not be forgotten that the shorter we make the sittings and the longer the intervals, so much the more slow and uncertain do we find the reaction, while the disease thereby gains in intensity and extent. If therefore, we aim at producing a rapid and free suppuration, the inhalations must be practiced as often and as long as possible, in quarter hour sittings every half hour, and, on the first and second day, three, or at the utmost four, hours of sleep must suffice for the patient, while nourishment must be supplied in small portions in the intervals between the separate sittings. Later on, when the pseudo membranes have been partially cast off, as well as in certain lighter cases, hourly sittings of about a quarter hour's duration suffice, and a longer time—six or eight hours—can be allowed for sleep. Even when a complete separation of the membranes has taken place, so long as a secretion of pus is still perceived, inhalations should be practiced every two or three hours."

As a means of prevention of general systemic poisoning, persistent rinsing and gargling of the mouth and throat is of great service. In this way decomposing substances and septic ferments are destroyed or washed away. For this purpose long arduous experimentation has determined a solution composed of alcohol, one part, and diluted chlorine water, three parts, to be preferable to all others. Next in order stands permanganate of potash in solution, one and one-half to two and a half grains to the ounce of water, and a solution of carbolic acid of same strength. The remedy, which ever is selected, should be used at least every hour. Where, from extreme youth or other cause, the patient is unable to cleanse his mouth, an attendant should do so by the use of a syringe. It should not, however, be forgotten that a complete destruction of the micrococcus is impossible even with the most industrious use of this means, for the fungus may have already gained lodgment in the tissues of the mucous membrane, the serous canaliculi and lymphatic vessels, as it assuredly exists and grows in the thick brawn-like deposit. Nor can the means just indicated see any limit to the inflammation. The most certain process of prevention of the toxic influences is to as completely as possible separate the diseased tissue from the healthy, and this can only be attained by establishing a rapid and abundant suppuration, forcing a line of demarkcation by assisting nature through the capacity for reaction which belongs to the affected tissues. The cleansing of the cavities with the solutions named is, however, a valuable adjunct.

This article is already too long, and I can say nothing of certain modifications of treatment when the diphtheritic process has invaded the nasal cavity, larynx, trachea and the bronchi; nor of the general treatment, except that it must be supporting to the system and prompt combative to complications. My only object is to invoke the philosophy of medicine in the treatment of diphtheria, as determined by pathological and physiological considerations. The million "specifics" have in no way stood the test of experience. The results of empirical treatment are simply terrible and must be relegated to fields of barbarism. Empirical hnowledge, individual and statistical, gained by long and fearful ex-derience, is in testimony of the views here presented.—*Detroit Lancet.*

